# Prefrontal networks dynamically related to recovery from major depressive disorder: a longitudinal pharmacological fMRI study

**DOI:** 10.1038/s41398-019-0395-8

**Published:** 2019-02-04

**Authors:** Bernhard M. Meyer, Ulrich Rabl, Julia Huemer, Lucie Bartova, Klaudius Kalcher, Julian Provenzano, Christoph Brandner, Patrick Sezen, Siegfried Kasper, Alan F. Schatzberg, Ewald Moser, Gang Chen, Lukas Pezawas

**Affiliations:** 1 Division of General Psychiatry, Department of Psychiatry and Psychotherapy, Medical University of Vienna, Vienna, Austria; 2 Department of Child and Adolescent Psychiatry, Medical University of Vienna, Vienna, Austria; 3 MR Centre of Excellence, Medical University of Vienna, Vienna, Austria; 4 Center for Medical Physics and Biomedical Engineering, Medical University of Vienna, Vienna, Austria; 5 Department of Psychiatry and Behavioral Sciences, Stanford University School of Medicine, Palo Alto, CA USA; 6 Scientific and Statistical Computational Core, National Institute of Mental Health, Bethesda, MA USA

## Abstract

Due to lacking predictors of depression recovery, successful treatment of major depressive disorder (MDD) is frequently only achieved after therapeutic optimization leading to a prolonged suffering of patients. This study aimed to determine neural prognostic predictors identifying non-remitters prior or early after treatment initiation. Moreover, it intended to detect time-sensitive neural mediators indicating depression recovery. This longitudinal, interventional, single-arm, open-label, phase IV, pharmacological functional magnetic resonance imaging (fMRI) study comprised four scans at important stages prior (day 0) and after escitalopram treatment initiation (day 1, 28, and 56). Totally, 22 treatment-free MDD patients (age mean ± SD: 31.5 ± 7.7; females: 50%) suffering from a concurrent major depressive episode without any comorbid DSM-IV axis I diagnosis completed the study protocol. Primary outcome were neural prognostic predictors of depression recovery. Enhanced de-activation of anterior medial prefrontal cortex (amPFC, single neural mediator) indicated depression recovery correlating with MADRS score and working memory improvements. Strong dorsolateral PFC (dlPFC) activation and weak dlPFC-amPFC, dlPFC-posterior cingulate cortex (PCC), dlPFC-parietal lobe (PL) coupling (three prognostic predictors) hinted at depression recovery at day 0 and 1. Preresponse prediction of continuous (dlPFC-PL: R^2^_day1_ = 55.9%, 95% CI: 22.6–79%, *P* < 0.005) and dichotomous (specificity/sensitivity: SP/SN_day1_ = 0.91/0.82) recovery definitions remained significant after leave-one-out cross-validation. Identified prefrontal neural predictors might propel the future development of fMRI markers for clinical decision making, which could lead to increased response rates and adherence during acute phase treatment periods. Moreover, this study underscores the importance of the amPFC in depression recovery.

## Introduction

Major depressive disorder (MDD) is highly prevalent leading to increased disability and mortality^1^. About two-thirds of all patients suffer from residual symptoms after first-line treatment with selective serotonin reuptake inhibitors (SSRIs)^2,3^. As a consequence, treatment optimization is common clinical practice resulting in a prolongation of disability and suicidal ideation^4,5^.

The clinical necessity of depression recovery (DR) stratification^6–8^ has propelled research of clinical^9^, genetic^10–12^, and neural predictors^13^. Several studies highlighted the role of the anterior medial prefrontal cortex (amPFC), as well as the anterior (ACC) and posterior cingulate cortex (PCC) in the prediction of DR after SSRI treatment^14–16^. Support is also provided by studies applying different treatment modalities^17–19^ and by research underscoring the importance of these brain regions in MDD pathobiology^20,21^. Beyond the amPFC, regions such as the anterior insula^13,22^, or dorsolateral PFC (dlPFC)^23,24^ were further suggested as predictors of DR.

Unfortunately, even a large body of cross-sectional imaging studies has failed to conclusively identify brain mechanisms responsible for DR^7,8,13,25,26^. This lack of consistency is not surprising considering the temporal dynamics of interactions between mentioned brain systems^13^ affecting the individual outcome over the course of illness^27,28^. One promising approach assessing such longitudinal interactions is to study neural mediators and prognostic predictors of DR, which have hardly been investigated so far^13,25^ despite their obvious clinical implications^6,8,13,29,30^. In this context, a prognostic predictor is defined as a treatment- and recovery-preceding, cross-sectional imaging characteristic related to DR^13,30,31^. Hence, it is capable of identifying nonresponders prior any clinical sign of improvement. Consequently, it is uncorrelated to changing neuroimaging measures along recovery and thus time-invariant^12,13^. A neural mediator, however, exhibits typical changes that might reflect neural processes unfolding in concert with recovery from depression^13,30,31^. Hence, it is per definition a time-sensitive imaging measure correlating with DR^12,13^. Importantly, predictors based on longitudinal data differ substantially from a single predictor originating from a cross-sectional study design lacking any distinction between time-sensitive (mediator) and time-invariant (prognostic predictor) brain processes^13,27,32,33^. As a critical clinical consequence, validated prognostic predictors would provide objective markers of nonresponse that are available prior treatment initiation, whereas mediators support decisions of clinicians, researchers and drug developers along treatment^13^. Previous studies^34–36^ investigated unspecific “prognostic predictors”^31^ to foresee nonresponse across treatment groups. In contrast to “prescriptive predictors”^31^ of treatment-specific outcomes, these findings might improve our understanding of mechanisms involved in a suboptimal DR that are not targeted by current treatments to propel future developments.

The primary goal of this exploratory, longitudinal, pharmacological functional magnetic resonance imaging study (phMRI) was to determine neural prognostic predictors of DR. Moreover, we expected to gain insights into the temporal dynamics of DR by assessing neural mediators^13,27,28,33^ along treatment. Previous clinical^37–39^ and imaging studies^40–42^ provide compelling evidence that highly persistent cognitive symptoms such as memory deficits or rumination are related to unfavorable illness course in terms of onset, DR, chronicity and future relapse. However, a more direct cognitive measure is required for a clinical application^41^. The frequently used *n*-back working memory (WM) task^43^ is well suited to assess these underlying cognitive functions as the interaction between networks of cognitive control (e.g., the dlPFC/fronto-parietal control network)^42,44^ and emotional processing (e.g., the amPFC/default mode network, DMN)^40,45^. Particularly the *n*-back task-negative DMN^46^ has been less prone to artifacts as compared to other standard paradigms applied in MDD research^47,48^. Escitalopram was chosen as treatment, because it is the most-selective^49^ and most-prescribed SSRI worldwide^2,3^. During this 9 weeks lasting clinical trial, all 22 MDD patients underwent 4 scanning sessions resulting in a total of 88 functional acquisitions. Four scanning sessions were performed: at baseline (day 0, d0), after initial escitalopram treatment (day 1, d1), and twice in monthly intervals (day 28 and day 56), where a clinical response is expectable. First, we determined neural predictors of DR within an activation analysis. Next, we performed functional connectivity (FC) analyses for significant brain regions in order to investigate findings on a brain systems level. Finally, we assessed the impact of neural predictors of DR on cognitive performance and clinical parameters.

## Methods and materials

### Subjects

MDD outpatients were recruited at the outpatient clinic or by online and bulletin board advertisements. Patients were invited to the Department of Psychiatry and Psychotherapy at the Medical University of Vienna (MUV) to participate in this longitudinal, interventional, single-arm, open-label, phase IV phMRI study. Enrollment was under supervision of LP after a comprehensive clinical assessment including previous medical and psychiatric history, neurological, and medical examinations such as routine laboratory testing, electrocardiography, and blood pressure measurement. The following inclusion criteria were applied: (1) MDD diagnosis according to DSM-IV (German Structured Clinical Interview, SCID-I)^50^ and absence of any other axis I disorder, (2) Montgomery-Åsberg Depression Rating Scale (MADRS) score ≥20 and ≤30, (3) age between 18 and 50 years, (4) right-handedness, and (5) willingness to provide informed consent and ability to be managed as outpatient. Detailed exclusion criteria are listed in the supplement. Out of 26, 22 included patients completed the study protocol (Figure S1). Reasons for study dropout were: corrupted MRI data (*n* = 1), lacking adherence to the study protocol due to increased anxious distress (*n* = 2), and exclusion due to medical reasons unrelated to the study medication (*n* = 1). The study protocol was approved by the local Ethics Committee (1060/2010) according to the Declaration of Helsinki. Please note four deviations after trial registration: (1) less subjects enrolled due to lower drop-out rates, (2) inclusion of healthy controls (HC), (3) one scan (d28) added as we recognized the strength of a longitudinal design before enrollment, and (4) more importance attached to the MRI-outcome, because genetic effects are envisioned as small and below clinical importance^10^.

Patient recruitment, scanning and data analysis took place between 2011 and 2017. All patients underwent 4 MRI scanning sessions prior (d0), 4–8 h (d1), 4 (d28), and 8 weeks (d56) after escitalopram treatment initiation. Imaging data of gender- and age-matched HC were retrieved from a previously published cross-sectional study^40^ subserving as control group for untreated patients (d0). Escitalopram dosing reflected clinical practice with a fixed dose of 10 mg and the option to increase to 20 mg after d28 until the end of study visit in case of nonresponse (*n* = 9). The primary measure of DR was defined as percent change between pretreatment (d0) and end-of-treatment (d56) MADRS scores: DR = (1-MADRS_d56_/MADRS_d0_)*100. MADRS was utilized to calculate DR due to its superior sensitivity to symptom change and its dominant use in clinical trials investigating escitalopram^51^. Clinical variables and interviews including the Hamilton Rating Scale for Depression (HAMD-17) and Anxiety (HAMA), and the Clinical Global Impressions (CGI) scale were employed to evaluate clinical prognostic predictors of DR and to exclude confounding collinearity (Table 1; Tables S2 and S3).

**Table 1:** Comparison between clinical and imaging predictors of DR available at baseline (d0/d1)

Top predictors	${\bar{\mathrm x}}$ (s)/$\tilde x$ (Q_1_/Q_3_)	*R* ^2^	CI_95_	PRESS *P*_FDR_		Other assessments	${\bar{\mathrm x}}$ (s)/$\tilde x$ (Q_1_/Q_3_)
*Clinical information*						*Clinical informati* *on*	
TN (*n*)	12	14	0–47.3	19416^+^		Δ HAMD (%)	67 (32)
MDEi (*n*)	7	2.2	0–28.7	22471 n.s.		Δ HAMA (%)	63.3 (27.3)
Adolescent onset (*n*)	11	1.5	0–26.7	23055 n.s.		Δ CGI-S (%)	0.33 (0.17/0.5)
MADRS	27 (2.7)	15.8	0–49.1	24415^+^		HAMD	19 (4.3)
						HAMA	21.1 (4.9)
*Imaging measures*						CGI-S	6 (5/6)
dlPFC-PL_Σ(d0,1)_		59.1	26.4–80.8	10746*		2B accuracy	65.4 (24.1)
MV_Σ(d0,1)_		63	31.4–83	11423*		Age (years)	31.5 (7.7)
dlPFC-PL_d1_		55.8	22.6–79	11437**		Gender (*n* female)	11
MV_d1_		55.4	22.1–78.7	12692**		Education (years)	12.3 (1.13)
MV_d0_		37.6	6.6–67.5	12719**		Previous PT (*n*)	10
dlPFC_Σ(d0,1)_		53	19.6–77.4	12900**		Suicide attempts (*n*)	3
dlPFC-amPFC_d1_		46.9	13.8–73.8	13083**		WST	33.1 (4.5)

Top baseline clinical and imaging predictors of DR are indicated by lowest PRESS values (left column). Other assessed clinical characteristics (right column) including secondary outcome measures (**Δ** = % change d0–d56) are shown as mean (SD) or median (Q 1/3), where appropriate. All data were CV to improve generalizability and comparability between models. Imaging predictors outperformed higher baseline MADRS values and TN as the only trendwise, uncorrected clinical characteristics related to better DR. Secondary outcome measures (**Δ** = % change d0–d56) and less predictive clinical characteristics were presented (second column) as mean (SD) or median (Q 1/3), where appropriate. *P*, uncorrected *P* value; *P*_FDR_, false-discovery-rate corrected value; *P* < 0.005 corrected^**^, *P* < 0.05 corrected *, *P* < 0.10 uncorrected^+^; *2B* 2-back, *CI* confidence interval, *CV* leave-one-out cross-validation, *DR* depression recovery, *TN* antidepressant treatment naive, *HAMD/HAMA* Hamilton depression/anxiety rating scale, *MADRS* Montgomery-Åsberg depression rating scale, *MDEi* concurrent Major Depressive Episode is index episode, *MV* multivariate model, *n* number, *PT* psychotherapy, *PRESS* predicted residual error sum of squares, *Q* quartile, *R*^2^ variance, *SD* standard deviation, *d* day, *WST* Wortschatztest (German vocabulary scale), *amPFC* anterior-medial PFC, *dlPFC* dorsolateral PFC, *PL* parietal lobe, *PCC* posterior cingulate cortex

### Imaging

Subjects performed the *n*-back task comprising two levels (0-back, 2-back) in each of the four longitudinal MRI sessions. Longitudinal WM performance defined^40^ as percent correct responses (2-back accuracy, %) was correlated with clinical and imaging data (Fig. 2b, Table S2). Data from a 3 T Siemens TRIO scanner (12-channel standard head coil, Siemens Healthcare Systems, Germany) was preprocessed with AFNI (http://afni.nimh.nih.gov/afni/) implemented into an R framework (http://cran.r-project.org/), as described previously^40^ and in the supplement.

### Local activation

Second-level analysis of longitudinal activation data utilized a linear-mixed effects model (3dLME) that included first-order autocorrelations between consecutive sessions^52,53^. To find time-invariant prognostic predictors of DR, we calculated the DR main effect on neural activation across scan sessions^13,30,31^. To detect time-sensitive neural activation that could mediate DR, the interaction-term of DR and scan session was calculated^13,30,31^. All computed models further included age, gender, and scan session as nuisance variables. Random effects were defined for intercept and slope across scan sessions to improve generalizability^54,55^.

### Context-dependent and context-independent functional connectivity

**Fig. 1:**
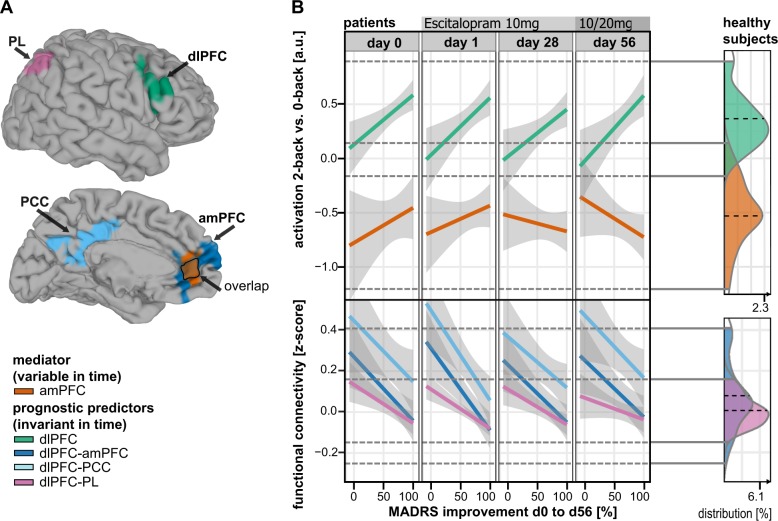
Neural mediator and prognostic predictors of depression recovery. **a** Clusters of significant prognostic value (FWE corrected) for DR (*n* = 22). **b** Neural mediator: Enhanced amPFC (orange) de-activation indicates improvements of depressive symptoms. Prognostic predictors: All four scans showed comparable results even weeks ahead of initial clinical response. Stronger dlPFC activation (green) accompanied by weaker dlPFC-amPFC (blue), dlPFC-PCC (cyan), and dlPFC-PL (purple) functional connectivity predicts beneficial depression recovery. Connectivity results for PL are related to 0-back conditions and therefore context-dependent. Healthy subjects: Density plots (right column) demonstrate no significant difference of imaging measures between MDD patients and matched healthy controls (HC) when comparing baseline data on a cross-sectional basis. amPFC anterior medial prefrontal cortex, dlPFC dorsolateral PFC, PCC posterior cingulate cortex, PL parietal lobe, 2B-0B 2-back vs. 0-back contrast

Second-level analyses were performed on seed-to-voxel FC maps in analogy to our activation analysis by using 3dLME^52,53^. Context-independent FC analysis correlated time-series after removing task-evoked co-activations^40,47^. Psycho-physiological interaction (PPI) analyses mapped the integration of seed regions specifically during 0B and 2B conditions, respectively. Activation analyses identified two significant clusters that defined our seed regions of interest (ROIs: amPFC mediator/dlPFC prognostic predictor of DR; red/purple, Fig. 1a).

### Post hoc statistics and plots

**Fig. 2:**
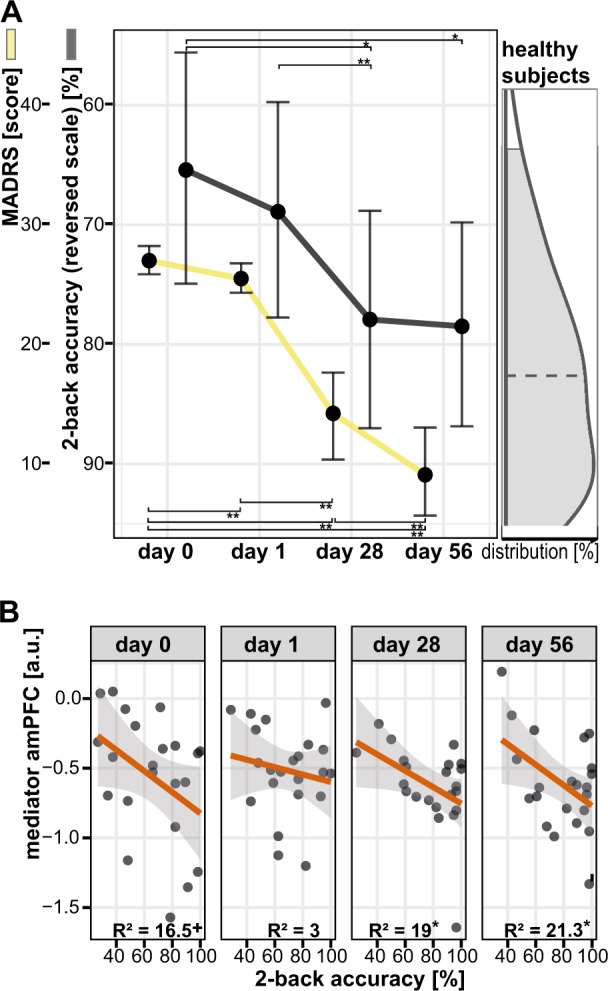
Working memory performance and mediator of depression recovery. **a** Depression symptoms (first *y*-axis) and n-back working memory performance (2B accuracy, %, second *y*-axis) improvements were strongest from day 1 to day 28, and tend to normalize compared to healthy subjects (right density plot). This might suggest working memory performance as cognitive correlate of depression symptoms. **b** A correlation of working memory performance and amPFC de-activation after first improvements (from day 28 to day 56) indicates that enhanced DMN suppression is beneficial for both, depression symptoms and working memory performance. ^+^ trendwise significant (*P* < 0.10), *,** significant (*P* < 0.05, 0.01), amPFC anterior medial prefrontal cortex, DMN default mode network, MADRS Montgomery-Åsberg Depression Rating Scale, R^2^ explained variance

**Fig. 3:**
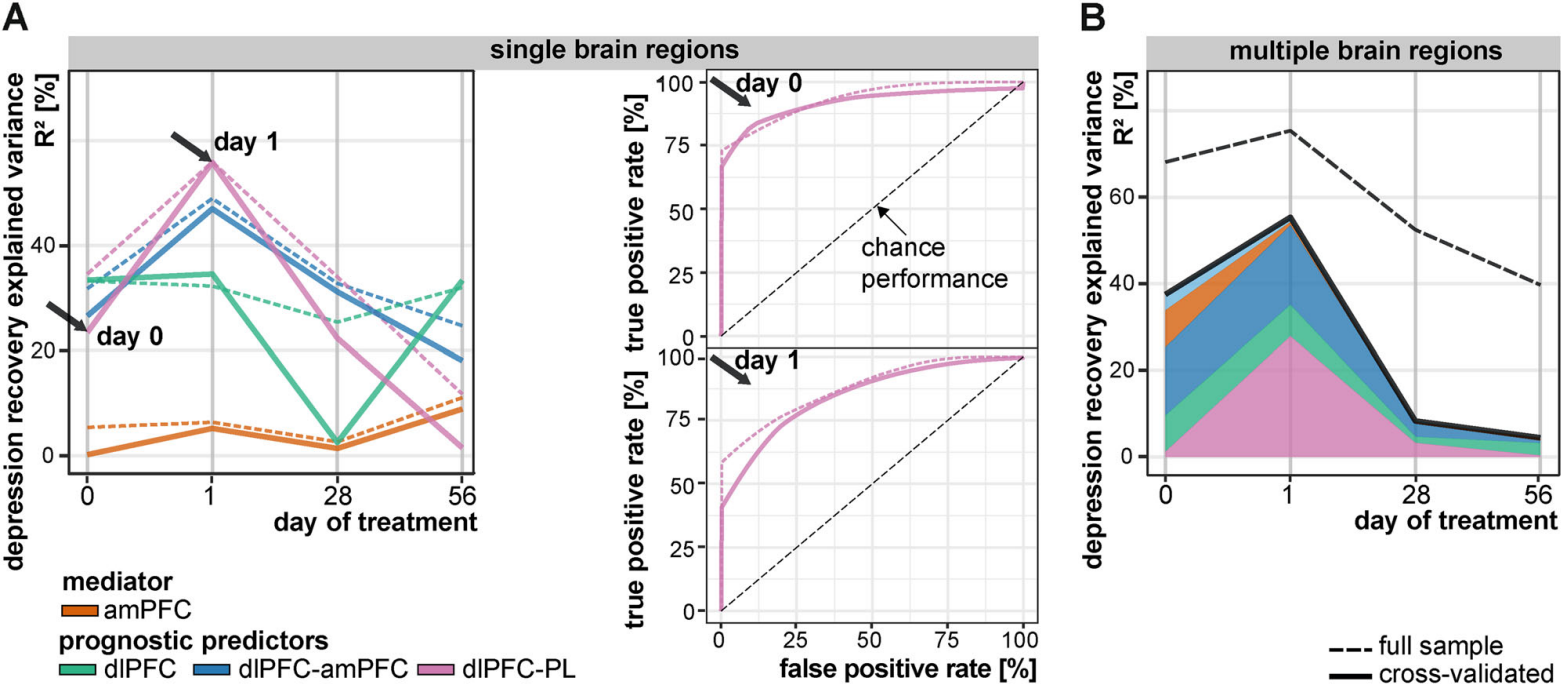
Diagnostic ability of imaging predictors to anticipate depression recovery: leave-one-out cross-validation (solid line, CV, *n* − 1 patients) vs. full sample effect size estimations (dashed line, *n* patients). **a** Explained variance remained large for all univariate models (single brain regions) prior clinical response (day 0 and especially day 1). ROC curves display the ability of the dlPFC-PL functional connectivity to differentiate also between dichotomous outcomes (remitters and non-remitters) prior a clinically observable response (day 0 and 1). Sensitivity (true positive rate) informs about correct remitter detection and specificity (1—false positive rate) about nonremitter detection. **b** Effect size remained similar for the multivariate model (all brain regions) after cross-validation. Overfitting, indicated by a large difference between dashed and solid lines, occurred predominantly using multivariate models at late trial stages (day 28 and day 56). This indicates shared information within this system of brain regions (for more details see Figures S4, S5 and Tables S3, S5). amPFC anterior medial prefrontal cortex, dlPFC dorsolateral PFC, PCC posterior cingulate cortex, PL parietal lobe, ROC receiver operating characteristics

Correlations between 2B accuracy, MADRS scores and all four imaging clusters were calculated (Fig. 2). DR outcome was primarily defined continuous to avoid power loss entailed by artificial dichotomization^56^. Still, clinical decision-making benefits from the prediction of dichotomized endpoints (MADRS_d56_). Hence, *post hoc* receiver operating characteristics (ROC, Fig. 3, Figure S5, Table S5) illustrate the prediction of a dichotomous endpoint defined as the median-split corresponding to MADRS_d56_ values ≤5 for remitters and ≥10 for nonremitters and complying with clinical cut-offs for remission^57^. Leave-one-out cross-validation (CV) was applied to improve generalizability, comparability and to avoid overfitting^25^ (Table 2, Table S5). Statistics were prepared in R 3.1.2 (http://cran-r-project.org/) on extracted means of significant clusters (*P* < 0.05 corrected, two-tailed). All models combining several predictors (Fig. 3b and Figures S4 and S5) were based on a uniformly weighted sum of values that were scaled and centered before. This additive score is envisioned as more intuitive and robust than using optimized weights for each predictor that likely entail overfitting^58^.

**Table 2:** Neural mediator and prognostic predictors of depression recovery

Region	BA	Cluster (mm^3^)	*t* (d*f*)	*z*	*P* value	*x*	*y*	*z*
***Mediator***								
amPFC	32, 24	2521	−3.41 (64)	−2.92	0.001^**^	−6	38	10
***Prognostic predictors***								
dlPFC	46, 9	3907	6.06 (18)	4.26	<0.001^**^	45	16	19
dlPFC-PCC	10	6859	−6.16 (18)	−4.31	<0.001^**^	10	−57	30
dlPFC-amPFC	24	5903	−5.52 (18)	−3.99	<0.001^**^	6	29	12
dlPFC-PL	39	3813	−5.86 (18)	−4.17	<0.001^**^	34	65	38

*x*, *y*, *z* are coordinates in Talairach space (LPI). Family-wise error rate (FWE) corrected *P* < 0.005^**^, 2B 2-back, 0B 0-back, BA Brodmann area, FC functional connectivity, amPFC anterior-medial PFC, dlPFC dorsolateral PFC, PL parietal lobe, PCC posterior cingulate cortex

## Results

### Demographics, clinical characteristics, and predictors

A total of 22 adult MDD patients (22–46 years; mean ± SD = 31.5 ± 7.7; 50% females) with a concurrent major depressive episode completed the study protocol. A total of 66 HC were exactly matched for patients’ gender (50% females), but were significantly younger also due to the relatively large number of HC (22–43 years; mean ± SD = 26.3 ± 3.4; *t*(24) = 3.1, *P* = 0.005). Patients (Table 1) were unmedicated for at least two months at d0 and suffered predominantly from a moderate MDE (27% mild, 55% moderate, 18% severe)^59^. A large proportion of patients was antidepressant naive (41%) and had never received previous psychopharmacological (50%) or psychotherapeutic treatment (55%). Clinical predictors revealed a trend towards better DR for patients with no previous antidepressant treatment (Table 1, left). Remaining baseline characteristics including WM performance did not predict later DR.

### Neural mediator of depression recovery and behavioral correlate

The recruitment of neural networks during task performance was comparable to previous reports^40^ (Supplement, Figure S2, Table S1). Neural mediators of DR were defined^13,30,31^ as time-sensitive interaction effects of DR on brain activation for all scan sessions (d0, d1, d28, and d56). One cluster comprising the amPFC reached statistical significance (orange, Fig. 1, Table 2). After clinical response, enhanced de-activation in this region was predictive for later DR measured from baseline to end-of-study (Fig. 1). Moreover, amPFC de-activation was related to depression severity (Fig. 2a, Table S2) and WM performance improvements (Fig. 2b, *p*_d28,d56_ < 0.05). Average WM performance improved along with depression symptom remission mainly between sessions d1 and d28 and to a level comparable to HC (Fig. 2a, Table S2). This cannot simply be explained by training effects, which are expected to be maximally between d0 and d1 due to novelty effects. Activation changed but remained within the range observed in HC (Fig. 1, density plot).

### Neural prognostic predictors of DR

Neural prognostic predictors of DR were defined^13,30,31^ as the time-invariant main effect of DR for each scan session. Our analysis revealed one significant activation cluster encompassing the right dlPFC and parts of the adjacent anterior insula (green, Fig. 1, Table 2). Both preresponse sessions (d0, 1) predicted a beneficial DR in case of strong dlPFC predictor activation, as confirmed by conservative CV (Table 1, Table S3). Next, the amPFC and dlPFC clusters resulting from activation analyses were used as seeds for context-independent and context-dependent (PPI) FC analyses. Context-independent FC of the dlPFC seed revealed two significant clusters within the DMN: PCC and amPFC (lightblue/blue, Fig. 1, Table 2). Context-dependent FC analysis showed an interaction of dlPFC-PL integration and 0B activation in the parietal lobe (PL, purple, Fig. 1, Table 2). Hence, responders showed weaker dlPFC-PL integration during the 0B conditions and/or their dlPFC suppresses PL 0B activation. Across all session and predictors, a weaker FC of the dlPFC was found beneficial for DR while all values were within the range of HC (density plot, Fig. 1).

### Localization of effects in the amPFC

We noticed that the dlPFC seed revealed a predictive cluster in the amPFC (blue, Fig. 1a), but we found no cluster in the dlPFC or elsewhere after using the partly overlapping anterior-perigenual amPFC seed (orange, Fig. 1a) despite the undirected nature of FC analyses. Hence, we *post hoc* analyzed Harvard-Oxford atlas-defined^60,61^ masks (Figure S7) and spherical seeds^62^ (supplemental video). The atlas-defined subgenual seed revealed the largest prognostic predictor cluster in the dlPFC of all three averaged ACC/amPFC masks (see Figure S7). Still, the punctum maximum was located neither in the mediator region nor in the posterior subgenual ACC (see sACC and pACC labels in the video), but spread from perigenual (i6) to anterior subgenual ACC regions (i8).

### Cross-validation of neural predictors

The clinical significance and large effects^63^ of scans conducted prior to clinical response (d0, 1) as predictors of DR are highlighted by the conservative CV (Fig. 3a, Table 1, Table S3). Longitudinally, prognostic predictors tend to show the largest effects ahead of response (max. CV *R*^2^: dlPFC-PL FCd_d1_ = 55.8%). Combined with other consistency measures (Figure S3 CD; Table S4), this indicates that timing matters, and, statistically speaking, a low between-session interchangeability^64^. Incorporating multiple brain regions (Fig. 3b) and scans (Figure S4) improved minimal rather than maximal predictive performance, thus enhancing prognostic stability. Inclusion of motion nuisance and clinical variables did not alter these results (Table S3). As expected, model overfitting occurred predominantly for multivariate models after incorporating multiple brain regions according to differences between conservative CV and standard full sample results (solid vs. dashed lines, Fig. 3b).

Prediction of the dichotomous remission outcome (ROC after CV, Fig. 2, Figure S5) showed large effects for all univariate FC prognostic predictors at baseline (Table S5, area under the curve, AUC_d0,d1,Σ(d0,1)_ > 0.79). The dlPFC-PL prognostic predictor provides an optimal specificity and sensitivity trade-off (Youden index = SP + SN-1: SP/SN_d0_ = 1/0.73; SP/SN_d1_ 0.91/0.82) close to the clinically important specificity-optimized (SP_max_) cut-off, which maximizes the detection of nonremitters.

## Discussion

This study aimed to identify neural prognostic predictors to anticipate suboptimal DR at four important treatment stages prior and during antidepressant treatment. Moreover, we analyzed changes of neural mediators, which are thought to trace brain systems functionally and causally related to the later clinical outcome. Finally, we evaluated these markers in terms of clinical use by behavioral data analysis and conservative effect size measures.

Consistent with cross-sectional imaging studies^14,15,40^, we identified potential neural markers of DR. Enhanced amPFC de-activation (mediator) correlated with symptom alleviation and therefore DR. In contrast, stronger dlPFC activation accompanied by a weaker coupling between dlPFC-amPFC, dlPFC-PCC, and dlPFC-PL (prognostic predictors) indicated beneficial DR^23,24,40^. These prognostic predictors showed statistically and clinically significant effects predominantly prior to clinical response. The supplemental video shows the spatial distribution of all effects located in the ACC/amPFC.

The detected neural mediator suggests that changes of amPFC de-activation are crucial to mitigate depressive symptoms^40,45,65^ (Fig. 1) and, specifically, persistent residual cognitive impairments^41,66^ (Fig. 2b) after clinical response (d28, d56). Hence, the amPFC mediator might inform clinical decision-making at early stages of therapy^13,30^ underlining the critical role of the DMN in DR^13,40,45^. Changes in amPFC activation, a region with relatively high serotonin transporter density^67^, were also observed in human SSRI-challenge studies^46,68^ and correlated to serotonin reuptake velocity in platelets^69^. Previous longitudinal SSRI treatment studies described functional changes within the amPFC in MDD responders^14,19,70,71^. Interestingly in terms of MDD treatment-specificity, this brain region was not only related to recovery in studies investigating serotonergic compounds, but also other antidepressants^17^, placebo effects^72^, psychotherapy^19^, deep brain stimulation^18^, and sleep deprivation^73^. On a clinical level, the amPFC was previously associated with rumination by using the same *n*-back experiment^40^. This corroborates the notion that decreasing amPFC activation during externally oriented tasks (e.g., *n*-back) represents successful DMN suppression necessary for cognitive performance and style improvement^40,45,65,74^ crucial for DR^37^.

Contrasting the mediator, the ascertained prognostic predictors of DR were statistically significant ahead of initial clinical response (Fig. 1). Stronger dlPFC activation and a weaker context-independent (dlPFC-amPFC, dlPFC-PCC) and context-dependent coupling during 0B conditions (dlPFC-PL) showed favorable effects on DR across all four sessions. The “cognitive neuropsychological” model^42^ of depression and a rich body of imaging studies^46,69^ indicate that SSRIs target primarily medial rather than lateral regions in the PFC^46,75^. In line with this model, we observed dynamic changes in the medial PFC and persistent factors in the lateral PFC, although both were markers for DR. These regions putatively cooperate like a pilot (amPFC/mediator) communicating with the air traffic control tower (dlPFC activation and FC/prognostic predictors). If you treat pilots to help them reaching their target, you also need a sanity check of the interplay with the control tower. The brain system orchestrated by the dlPFC might form such an auxiliary top-down regulation system. The *n*-back task tests the capacity of this system by increasing the limbic bottom-up interference during less-demanding 0B conditions^42^ in analogy to challenging weather conditions in a flight simulator. This might explain the additional prognostic predictor during 0B conditions (dlPFC-PL FCd, Fig. 1). The pattern of brain regions involved in this system (dlPFC, PL, dorsal amPFC) can be associated with reappraisal, selective attention and distraction^76^. These are emotion regulation strategies that require patients to (A) actively control (B) a model-based change while (C) explicitly holding mental representations of their goal within their mind^77,78^. These cognitive capacities tested by *n*-back^79^ and other prefrontal batteries were clinically related to a suboptimal course of depression^80,81^ and might be important targets of complementary treatments^26,42,82–84^.

This study highlights advantages of longitudinal phMRI prediction studies^13,27,52^. However, statistical significance is not sufficient to inform clinical decision-making^8,29,85^. As desired in prediction, pre-response imaging markers (Figure S4 R2_d0,d1,Σ(d0,1)_) tend to show larger effects as compared to post-response imaging or also preresponse clinical^9^ markers. Prognostic predictors are conceptualized as time-invariant and uncorrelated to DR itself^12,13,29^. Nonetheless, relatively large multisession effects (Figure S4) and low between-session consistency (Figure S3AB, Table S4) suggest that all scans contribute unique information from distinct treatment phases with low interchangeability^64^. ROC analyses (Fig. 3) facilitate translation of continuous DR predictors to predictors of dichotomous remission with known statistical costs to support decision-making as desired by most clinicians^56^. These results (Fig. 3a) suggest that an automatized measure (dlPFC-PL FCd) obtained by a single non-invasive 15-min fMRI is capable to improve patient stratification^86^. The threshold of clinically acceptable misclassifications might be larger in this context compared to, e.g., screening of orphan diseases, because it critically depends on two factors. Firstly, low economic and safety risks of (falsely) initiated second-line treatments would favor ordering the test, which would remain to positively influence clinical decision making^8^. Secondly, the pretest probability is favorably high due to a moderate antidepressant treatment response rate of 50–60%^5,87^.

This study has several limitations. Considering that secondary analyses are nonindependent from voxel-wise analyses, effect sizes likely remain inflated even after CV due to circularity^88^. Clinical prediction studies typically include CV, which is the necessary tough not sufficient step before clinical translation. This step is rarely applied in imaging prediction studies^25,89^ although the out-of-sample model could dramatically change results, as shown in our study (e.g., Figure 3). Still, our results primarily apply to noncomorbid, moderately depressed, nonsuicidal, and rather young adult patients without any history of previous treatment-resistance. Hence, results in comorbid, adolescent, geriatric, suicidal, therapy-resistant or -refractory MDD samples might differ substantially^90^. Since the intention of this study was to investigate prognostic predictors and mediators in a real-world clinical scenario with higher external validity and not the efficacy of the most prescribed antidepressant^2,3,75^, we employed an open-label study design without any placebo control, randomization, or blinding. Given the existence of intermixed placebo and drug effects in daily clinical practice^8^, neural predictors and mediators are explicitly categorized as nonspecific prognostic and not prescriptive predictors of DR^29,31,87^. However, we feel confident that placebo effects did not severely confound our interpretation, since we focused primarily on the clinically more important group of nonresponders, in which no placebo response is expected given the absence of any relevant overall treatment effect. Further, in contrast to between-group designs (MDD vs. HC, Fig. 1), within-group designs, and, particularly, predictive studies intend to utilize heterogeneity in neural patterns to reliably stratify patients without necessarily understanding the underlying nosology and etiology^6,7,25^.

Summarizing, our phMRI study characterized the longitudinal dynamic of neural prognostic predictors and a mediator candidate of DR. Enhanced de-activation of the amPFC (mediator) in remitters underscores the importance of the DMN in DR. Weaker activation and stronger FC of the dlPFC (prognostic predictors) was related to insufficient DR, which highlights the possibility to identify MDD non-remitters prior to treatment initiation. If replicated, these data encourage the clinical use of fMRI for individual risk prediction of a suboptimal illness course, which is urgently needed given the present insufficient sequential treatment algorithms.

## Supplementary information

Supplemental Text

Supplemental Video
