# Supplementary material for: Prefrontal networks dynamically related to recovery from major depressive disorder: a longitudinal pharmacological fMRI study
Source: Transl Psychiatry. 2019 Feb 4;9:64. doi: 10.1038/s41398-019-0395-8 (PMC6362173; doi:10.1038/s41398-019-0395-8)
Supplement: Supplementary file 1 — Supplemental Text [file 41398_2019_395_MOESM1_ESM.docx]

**­­**

**Online-Only**

**Supplemental Information**

## **Methods**

*Patients*

Exclusion criteria were defined as follows: 1.) previous or concurrent major medical or neurological illness or clinical significant alterations of laboratory measures, ECG, or physical exams 2.) QTc interval >450 msec (males) or >470 msec (females), 3.) contraindications to MRI, 4.) current pregnancy or breast feeding, 5.) suicidality, 6.) previous diagnosis of any major axis I disorder other than MDD as primary diagnosis such as schizophrenia, schizoaffective disorder, bipolar disorder, or addiction disorders except nicotine dependence and caffeine abuse, 7.) any continuous psychotropic drug use within the last two months prior study inclusion, 8) history of antidepressant non-response, 9.) history of drug allergy or hypersensitivity to escitalopram, and 10.) incompliance to the study protocol or instructions o the investigating team.

Depressive symptoms were assessed weekly during this eight weeks (56 days) lasting clinical trial as well as on the days of image acquisition (Flow Diagram in Figure S1). Escitalopram plasma samples were collected at days of scanning (d1, d28, d56) in order to verify the presence of therapeutic plasma levels as well as compliance related to drug intake in all patients. Clinical characteristics with skewed distribution were dichotomized (e.g. treatment naive, yes or no) to prepare linear modeling with depression recovery (DR) as dependent variable (Table 1). Adolescent onset was defined as MDD onset before age 19 (1).

*fMRI Paradigm*

All patients underwent standardized MRI safety instructions and a training for the employed *n*-back task to reduce potential habituation effects(2). Briefly, patients had to recall any number (1-4) seen two presentations before (2-back condition, 2B) or alternatively had to identify within a control condition the currently seen digit (0-back condition, 0B). This fMRI paradigm consisted of four 2B and four 0B blocks in total, each lasting for 30 seconds. Fourteen digits were pseudo-randomly presented for one second and followed by a second inter-stimulus-interval by using standard software (Presentation 10.3, http://www.neurobs.com/).

Working memory performance data were recorded using a button response box for each condition and assessed as percent correct responses (2B accuracy, %) (1, 3, 4). Subjects replied standardized with their right hand using the middle finger for digit 1, the ring finger for digit 2, the index finger for digit 3, and the little finger for digit 4. Longitudinal performance data were further related to corresponding depression severity data within a general linear model (Table S1, Figure 2). Associations were evaluated by the use of paired t-tests comparing any session relative to the pre-treatment visit (d0) with the goal to detect any association between working memory performance and depressive symptom improvements along the clinical trial.

*Motion handling*

To handle the potential influence of motion confounders, we applied a recommended (5, 6) two-steps strategy. This strategy was implemented before in a challenging developmental imaging-genetics study (7), which involved a typically more active adolescent subsample and smaller effects of interest. First, the preprocessing pipeline includes regression procedures (ANATICOR) to handle motion and scanner artifacts in terms of functional connectivity analyses as described above. Second, we calculated the 3D maximum displacement (x, y, z)(5) and observed moderate motion in all scans (maximum translation $\tilde{x}$ (Q_1_/Q_3)_: 0.49 (0.32/0.78) with, as expected (8), the highest value of 2.1mm in session d1 (4-8 hours after first medication). To prevent a potential sample selection bias by removing subjects with strong motion potentially related to anxiety and jitteriness symptoms at early stages (d1) of therapy, we decided to not remove sessions but to model the influence of motion. These cross-validated models revealed no influence of maximum translation on effect size estimates (see Table S3).

*Multiple Comparison Correction*

Both, activation and FC second-level results were corrected for multiple comparisons using family-wise error (FWE) in a whole-brain analysis of regions activated by the task (p < 0.01 uncorrected; 3dClustSim: iterations n = 10,000, voxel-wise threshold p = 0.01, dimensions: 74x87x69 grid, 2.19x2.19x2.19 mm³, a minimum cluster size of 130 voxels yielded a corrected p value of 0.05, smoothness estimated with 3dFWHMx). Given the two seeds were used for three types of FC analysis (simple without task, PPI 0B condition, PPI 2B condition) these corrected p values of clusters found in all three FC analyses were Bonferroni corrected across all six tests (cluster size of 266 voxels for required p of 0.008).

*Post-hoc Statistics for Extracted Mediator and Moderator values*

Reported inference and adjusted R² results shown in Figure 2 were calculated using the general linear model in R 3.1.2 and the PMVD (“proportional marginal variance decomposition”) metrics of the package ‘relaimpo’ (“relative importance metrics”) for proportional variance estimation. Please note, that the PMVD metrics was preferred against LMG (named after the authors Lindeman, Merenda, and Gold) due to its better interpretability for prediction(9).

Receiver Operating Characteristic (ROC) curves (two clinical scenarios, Figure 2; additional scenarios, Figure S5) were estimated with a log-concave smoothed bootstrap of 1000 iterations utilizing the pROC package (10).

*Comparisons and Plots*

The R-package ‘ggplot2’ (http://ggplot2.org/) was utilized to create plots. All inference, adjusted variance (R²) estimations, group comparisons, bootstrap simulations and correlation analyses were calculated in R 3.2.5 (http://cran-r-project.org/). Significant clusters after correction for multiple comparisons (p < 0.05, two tailed) were visualized on an averaged anatomical template.

## **Results**

*Working Memory Performance*

Working memory performance (Figure 2, Table S2) improved significantly from the first drug-free scan session (d0) to d28, as well as to d56 of treatment, but not between d0 and d1. This lack of performance difference supports the notion that the observed association between MADRS and working memory performance was not driven by task novelty or training effects given the considerably shorter time interval between d0 and d1 (< 1 week) compared to d1 and d28 as well as d28 and d56.

*Test-retest stability and Intra-Class-Correlations (ICC)*

Intra-class-correlations (ICC) were calculated to provide a sensitive measure of between-subject variance and to estimate the ability to distinguish individuals relative to each other. ICC values were mostly fair to good above 0.4 (Table S4). Compared to the literature, results for functional connectivity are highly similar(11) and for activation only slightly lower(12). This was to be expected because our study design intentionally triggered differences between sessions during antidepressant recovery as displayed in Figure 2.

*Recruitment of Neural Networks during Task Performance*

To demonstrate a comparable engagement of task-positive and task-negative neural networks to previous studies in our study sample, we calculated the working memory main effect (2B-0B) comprising all 88 available scans (Figure S2, Table S1). As expected we found the task to recruit the task-positive fronto-parietal control network (FPC) and the cingulo-opercular salience network (SA) (13, 14), which are thought to be related to working memory retrieval, maintenance and attention. Additionally, the employed *n*-back task recruited also core regions of the task-negative default mode network (DMN), which is envisioned to be relatively suppressed during external demands (14, 15), and highly relevant for working memory performance (16, 17) (Figure 2B). The FPC comprised dorsolateral prefrontal cortex (dlPFC), ventrolateral PFC (vlPFC), and the parietal lobe, the SA activated the dorsal anterior cingulate cortex (dACC) and the opercular dorsal anterior insula (AI). Deactivated cortical core regions of the DMN were the anterior medial prefrontal cortex (amPFC), adjacent anterior cingulate cortex (ACC), posterior cingulate cortex (PCC), as well as temporal regions and the temporo-parietal junction (TPJ).

*Post-hoc Effect Size*

Receiver operating characteristics (ROC) curves (Figure 3, Figure S5) and the predictive value (Table S3) of the imaging mediator and all prognostic predictors were calculated for two possible clinical scenarios: (A) use of single scans along ET and (B) use of multiple scans at a specific time-point. ROC parameters (Table 3, Table S5) based on the Youden index(18) as best trade-off between sensitivity and specificity were calculated for these clinical scenarios with a dichotomous DR outcome to evaluate detection of non-remitters despite its statistical disadvantage compared to continuous outcome.

## **References**

1. Bartova L, Meyer BM, Diers K, Rabl U, Scharinger C, Popovic A, et al. Reduced default mode network suppression during a working memory task in remitted major depression. J Psychiatr Res. 2015;64:9-18.

2. Callicott JH, Mattay VS, Bertolino A, Finn K, Coppola R, Frank JA, et al. Physiological characteristics of capacity constraints in working memory as revealed by functional MRI. Cereb Cortex. 1999;9(1):20-6.

3. Rose EJ, Simonotto E, Ebmeier KP. Limbic over-activity in depression during preserved performance on the n-back task. Neuroimage. 2006;29(1):203-15.

4. Schoning S, Zwitserlood P, Engelien A, Behnken A, Kugel H, Schiffbauer H, et al. Working-memory fMRI reveals cingulate hyperactivation in euthymic major depression. Human brain mapping. 2009;30(9):2746-56.

5. Van Dijk KR, Sabuncu MR, Buckner RL. The influence of head motion on intrinsic functional connectivity MRI. Neuroimage. 2012;59(1):431-8.

6. Jo HJ, Saad ZS, Simmons WK, Milbury LA, Cox RW. Mapping sources of correlation in resting state FMRI, with artifact detection and removal. Neuroimage. 2010;52(2):571-82.

7. Meyer BM, Huemer J, Rabl U, Boubela RN, Kalcher K, Berger A, et al. Oppositional COMT Val158Met effects on resting state functional connectivity in adolescents and adults. Brain Struct Funct. 2016;221(1):103-14.

8. Sinclair LI, Christmas DM, Hood SD, Potokar JP, Robertson A, Isaac A, et al. Antidepressant-induced jitteriness/anxiety syndrome: systematic review. Br J Psychiatry. 2009;194(6):483-90.

9. Gromping U. Estimators of relative importance in linear regression based on variance decomposition. Am Stat. 2007;61(2):139-47.

10. Robin X, Turck N, Hainard A, Tiberti N, Lisacek F, Sanchez JC, et al. pROC: an open-source package for R and S+ to analyze and compare ROC curves. BMC Bioinformatics. 2011;12:77.

11. Cao H, Plichta MM, Schafer A, Haddad L, Grimm O, Schneider M, et al. Test-retest reliability of fMRI-based graph theoretical properties during working memory, emotion processing, and resting state. Neuroimage. 2014;84:888-900.

12. Plichta MM, Schwarz AJ, Grimm O, Morgen K, Mier D, Haddad L, et al. Test-retest reliability of evoked BOLD signals from a cognitive-emotive fMRI test battery. Neuroimage. 2012;60(3):1746-58.

13. Seeley WW, Menon V, Schatzberg AF, Keller J, Glover GH, Kenna H, et al. Dissociable intrinsic connectivity networks for salience processing and executive control. J Neurosci. 2007;27(9):2349-56.

14. Menon V, Uddin LQ. Saliency, switching, attention and control: a network model of insula function. Brain Struct Funct. 2010;214(5-6):655-67.

15. Buckner RL, Andrews-Hanna JR, Schacter DL. The brain's default network: anatomy, function, and relevance to disease. Ann N Y Acad Sci. 2008;1124:1-38.

16. Koshino H, Minamoto T, Yaoi K, Osaka M, Osaka N. Coactivation of the Default Mode Network regions and Working Memory Network regions during task preparation. Sci Rep. 2014;4:5954.

17. Piccoli T, Valente G, Linden DE, Re M, Esposito F, Sack AT, et al. The default mode network and the working memory network are not anti-correlated during all phases of a working memory task. PLoS One. 2015;10(4):e0123354.

18. Perkins NJ, Schisterman EF. The inconsistency of "optimal" cutpoints obtained using two criteria based on the receiver operating characteristic curve. Am J Epidemiol. 2006;163(7):670-5.

19. Margulies DS, Kelly AM, Uddin LQ, Biswal BB, Castellanos FX, Milham MP. Mapping the functional connectivity of anterior cingulate cortex. Neuroimage. 2007;37(2):579-88.

# **Supplemental Figures:**

**Figure S1. Flow Diagram**

Abbreviations: MRI, magnetic resonance imaging

**Figure S2. *N*-back Task Main Effect Within all Patients (n=22) and Scans (4)**

Surface maps displays working memory load-dependent activation increased in the dlPFC and the vlPFC, the anterior insula, and the inferior parietal lobule, as well as decreased in the amPFC and the PCC, medial prefrontal-, temporal and parahippocampal gyrus. Colorbar represents t-statistics. Abbreviations: dlPFC, dorsolateral prefrontal cortex; vlPFC, ventrolateral prefrontal cortex; amPFC, anterior-medial prefrontal cortex; PCC, posterior cingulate cortex.


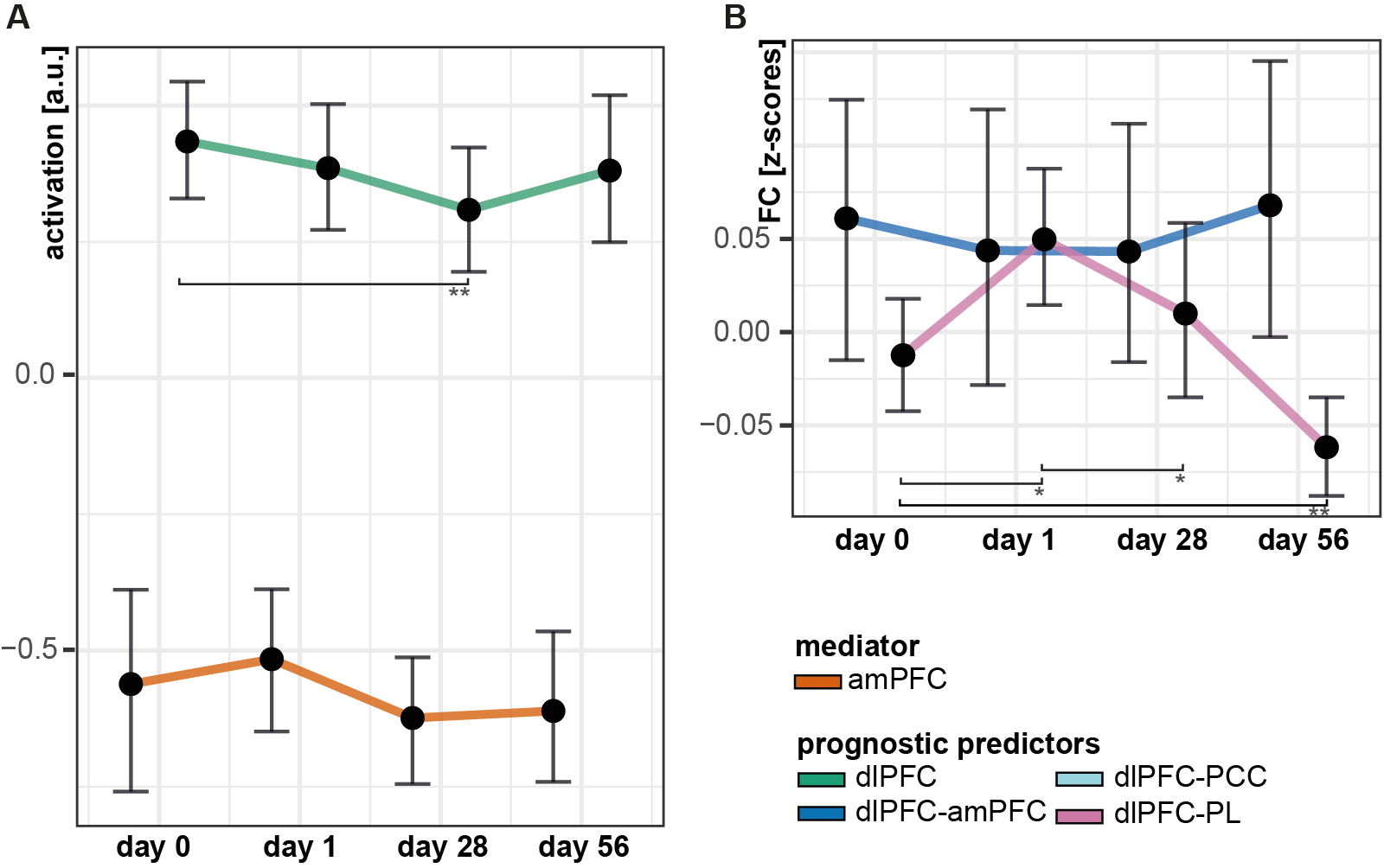


**Figure S3. Temporal Dynamics of Imaging Measures**

Temporal dynamics of activation (A) and functional connectivity (B) measures of brain regions related to depression recovery. The dlPFC activation shows potential training and/or depression severity effects, whereas the dlPFC-amPFC was the most stable cluster in terms of mean differences. Abbreviations: ^+^ trendwise significant (*P* < 0.10); ^*,**^ significant (*P* < 0.05, 0.01); 0 B, 0-back; 2B, 2-back; amPFC, anterior medial prefrontal cortex; dlPFC, dorsolateral PFC; PCC, posterior cingulate cortex; PL, parietal lobe; MADRS, Montgomery-Åsberg Depression Rating Scale; R^2^, explained variance

**
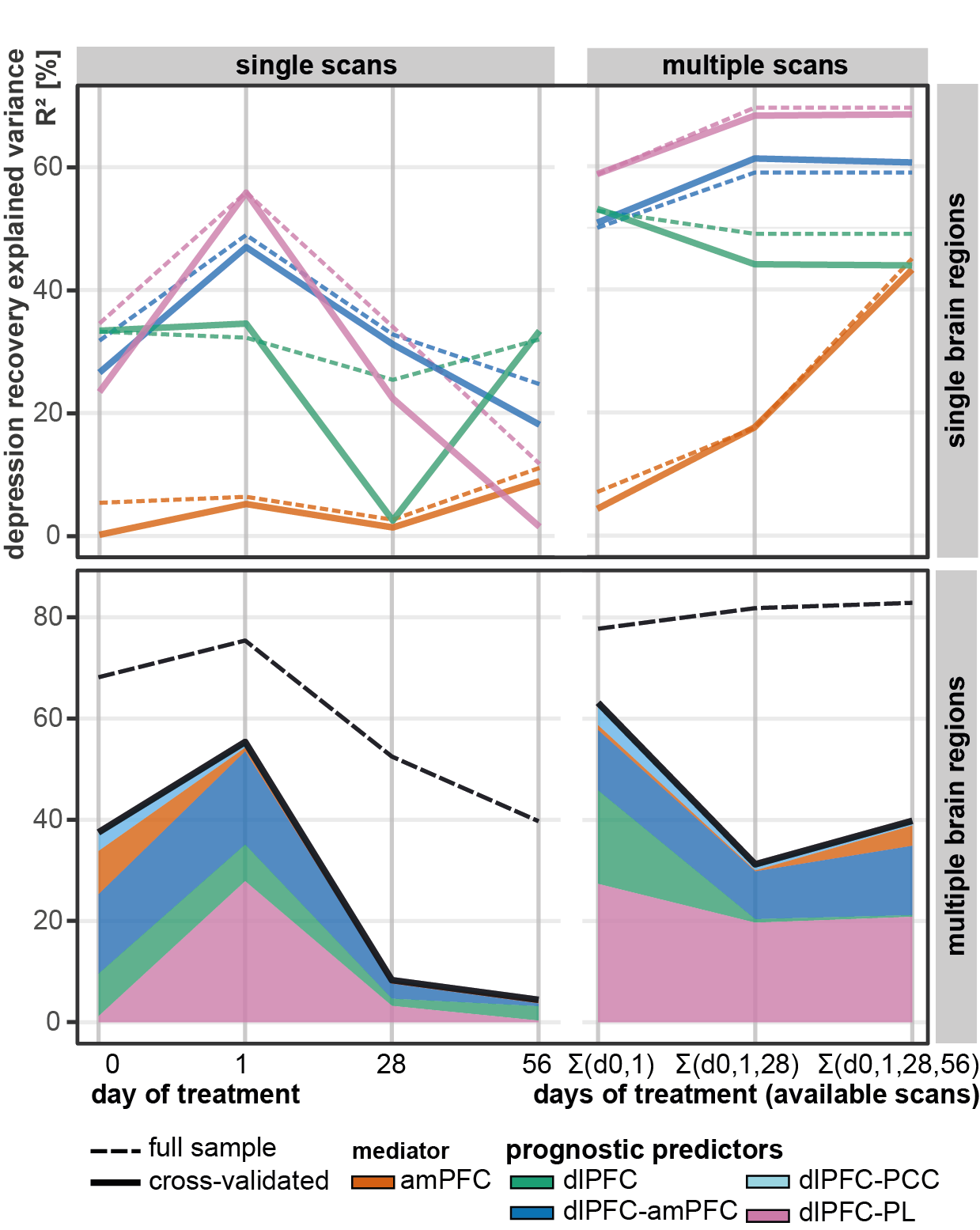
**

**Figure S4. Explained Variance of Depression Recovery**

Cross table displaying a comparison between leave-one-out cross-validation (solid line, CV, n-1 patients) and standard full model calculations (dashed line, n patients) including all variations not shown in the main manuscript (Figure 3). Columns compare single (left) vs. multiple scan sessions (right), whereas rows compare single (upper, univariate) vs. multiple predictors (multivariate). Performance values remained large for all models prior clinical response (day 0 and especially day 1). Overfitting, indicated by a large difference between dashed and solid lines, occurred predominately using multivariate models at late trial stages (day 28, day 56). Modeling multiple sessions and predictors did not improve maximum (but minimum) cross-validated performance and therefore predictive model stability. Redundant PCC plots are not displayed when mimicking the amPFC. Abbreviations: amPFC, anterior medial prefrontal cortex; dlPFC, dorsolateral PFC; PCC, posterior cingulate cortex; PL, parietal lobe

**
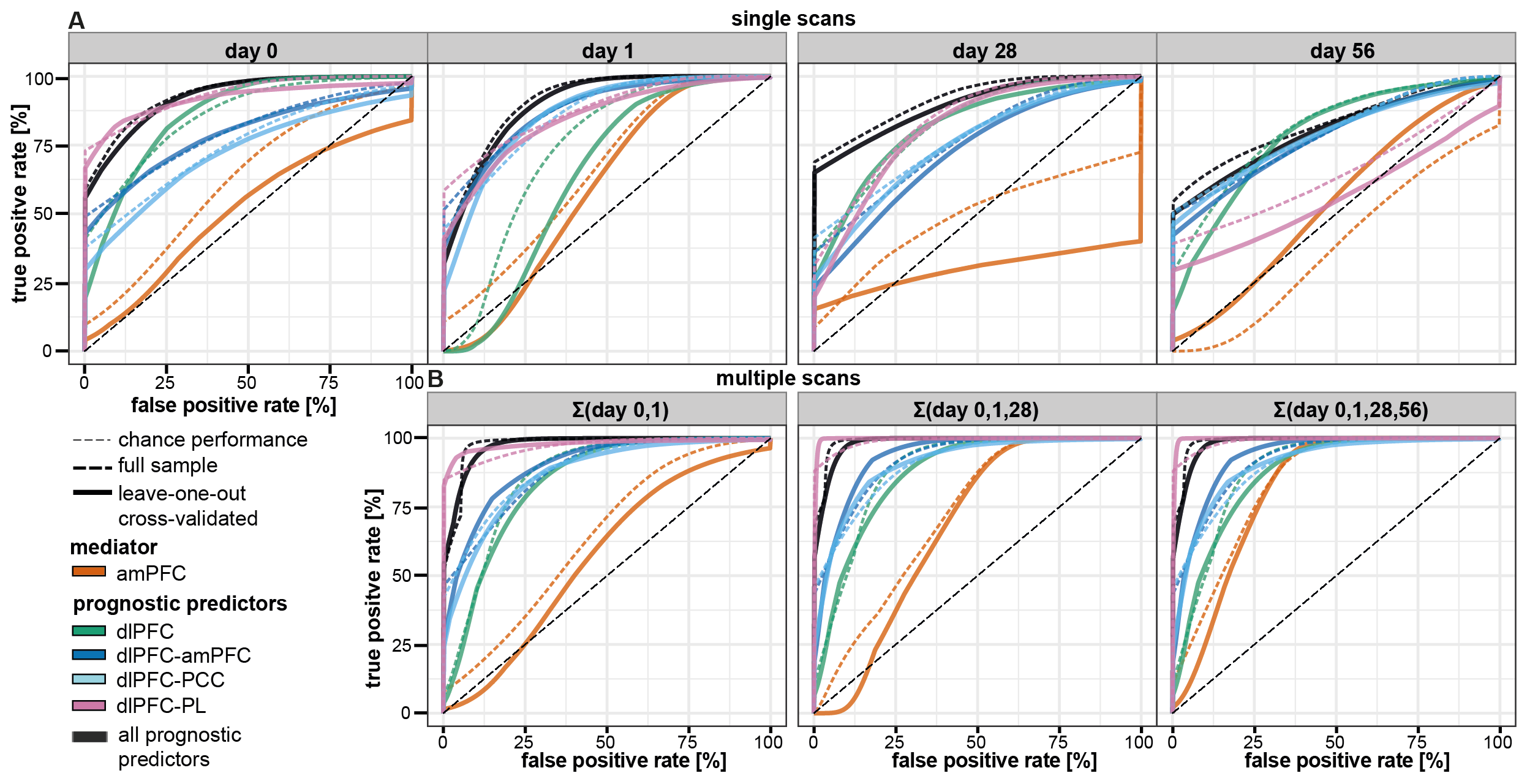
Figure S5. (Non-)Remission Detection by the Mediator and Prognostic Predictors**

Smoothed bootstrapped ROC curves were leave-one-out cross-validated for all mediators and prognostic predictors of dichotomized depression recovery outcome to detect non-remitters before clinical symptoms alleviation. Displayed ROC curves include also single and multiple scans not shown in the main manuscript (Figure 3). Sensitivity (true positive rate) informs about correct remitter detection and specificity (1 – false positive rate) about non-remitter detection. Abbreviations: amPFC, anterior medial prefrontal cortex; dlPFC, dorsolateral PFC; PCC, posterior cingulate cortex; PL, parietal lobe; ROC, Receiver Operating Characteristics


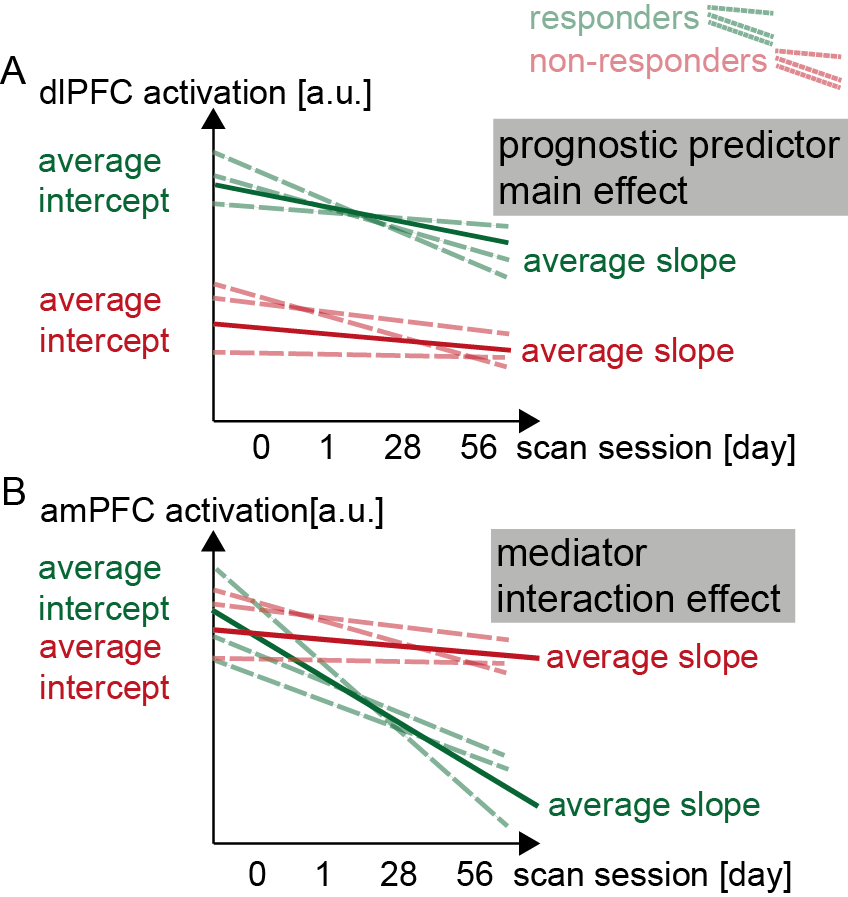


**Figure S6. Explanation and Illustration of the Statistical Model (3dLME)**

Two Linear-Mixed Effects models (3dLME) were applied: (A) First, the main-effect of DR across scan sessions was calculated to detect prognostic predictors. Our results indicated that stronger dlPFC activation (higher position, but parallel slope) was related to a favorable DR across scans. (B) Second, the interaction-term of DR and scan session was utilized to find different slopes across scan sessions. Our data of the amPFC revealed that the activation decreased (negative slope) stronger in patients with a beneficial outcome. Gender, age (nuisance variable) and DR were implemented as typically done in standard general linear models (GLM). Still, 3dLME additionally includes random effects for intercept and slope across sessions. This means that the activation at day 0 (intercept) and increase/decrease of activation across sessions (slope) were estimated for each patient individually (dashed lines) before effects can be summarized across patients (solid lines). This allows accounting for an individual treatment course to improve generalizability. Abbreviations: amPFC, anterior medial prefrontal cortex; dlPFC, dorsolateral PFC; DR, depression recovery


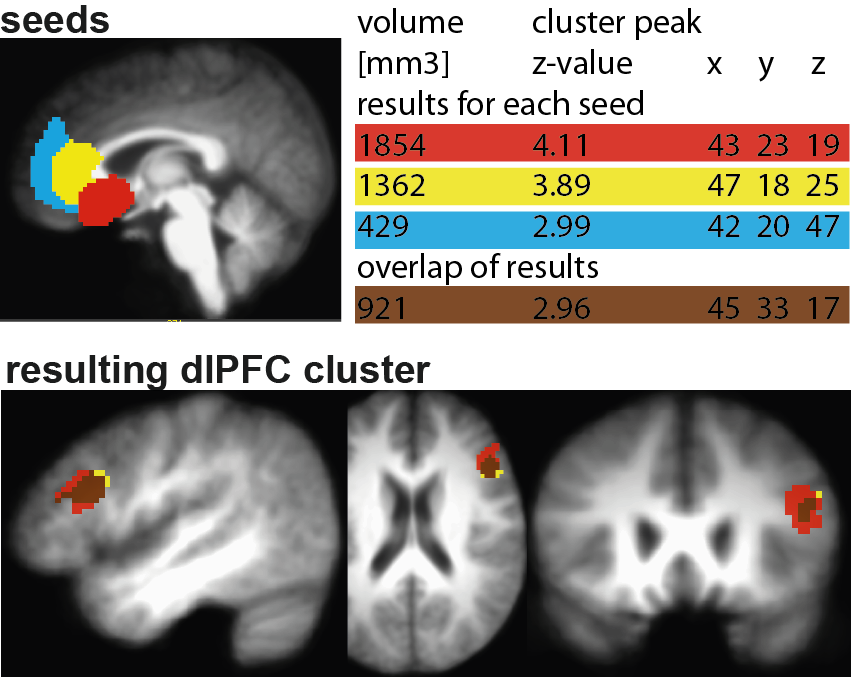


**Figure S7. dlPFC Results for Sub- and Perigenual amPFC Seeds**

The atlas-derived seed of the subgenual ACC (red) revealed stronger effects in terms of cluster size and peak z-values than the perigenual ACC (yellow) and the anterior ACC (blue) masks. This indicates that the averaged perigenual amPFC seed (mediator region) used in our main FC analysis did not reveal any significant results due to its relatively anterior location. x, y, z are coordinates in Talairach space (LPI). Abbreviations: family-wise error rate (FWE) corrected P <0.005**, 2B, 2-back, 0B; 0-back; BA, Brodmann area; FC, functional connectivity; ACC, anterior cingulate cortex; amPFC, anterior-medial PFC; dlPFC, dorsolateral PFC;

**Supplemental Video: ACC/amPFC Regions of Interest**

Mediator (orange) and prognostic predictor (green) effects reported in the main manuscript are displayed in the graph for subgenual, perigenual and anterior ROIs of the ACC/amPFC and indicated on the surface as white area. These ROIs served as *post-hoc* seed regions (yellow on medial surface) and its results were extracted from spheres located in the anterior-lateral (black) and posterior-medial dlPFC (blue = peak coordinates of activation prognostic predictor). Main ACC coordinates (i9-i5, s6-s7) were derived from Margulies et al., NI (2007)(19). Abbreviations: ACC, anterior cingulate cortex; amPFC, anterior-medial PFC; dlPFC, dorsolateral PFC; ROIs, regions of interest;

# **Supplemental Tables:**

| **Region** | **BA** | **Cluster (mm^3^)** | **z** | ***P*-value** | **x** | **y** | **z** |
| --- | --- | --- | --- | --- | --- | --- | --- |
| FPC and SN  IPL peak | 22,13 | 440755 | 13 | <0.001^**^ | 60 | -41 | 17 |
| DMN  PCC peak | 23,30,31 | 346179 | -11.6 | <0.001^**^ | -3 | -52 | 19 |
| DMN  TPJ peak | 39 | 3310 | -7.4 | <0.001^**^ | -49 | -61 | 25 |

**Table S1. Activation Main Effects of the *n*-back Task**

Positive z-value indicate an increase and negative a decrease of activation; x, y, z are coordinates in Talairach space (LPI). Abbreviations: FWE corrected *P* <0.005^**^; BA, Brodmann area; DMN, default mode network; FPC, fronto-parietal control network; IPL, inferior parietal lobe; PCC, posterior

cingulate cortex; SA, cingulo-opercular salience network; TPJ, temporo-parietal junction.

| **MADRS** | **d_0_** | **d_1_** | Δ(**d_0_,d_1_) (%)** | **d_28_** | Δ(**d_0_,d_28_)**  **(%)** | Δ(**d_1_,d_28_)** | **d_56_** | **Δ(d_0_,d_56_)**  **(%)** | Δ(**d_28_,d_56_)** |
| --- | --- | --- | --- | --- | --- | --- | --- | --- | --- |
| MADRS | 27  (2.7) | 25.5 (2.8) | *94.6^*^*  *(7.9)* | 14.2 (8.9) | *52.4^**^*  *(31.3)* | *^**^* | 9.1  (9) | ***33.1^**^***  ***(32.3)*** | *^**^* |
| HAMD | 19.1 (4.3) | 17.4 (4.1) | *92^*^*  *(13.5)* | 9.9  (4.7) | *52.9^**^*  *(27.2)* | *^**^* | 6.2  (5.1) | ***33.4^**^***  ***(26.6)*** | *^**^* |
| HAMA | 21.1  (4.9) | 19.3  (5.1) | *91.9^*^*  *(11.9)* | 10.5  (5.4) | *50.5^**^*  *(22.3)* | *^**^* | 7.9  (6.3) | ***36.5^**^***  ***(27.3)*** | *^*^* |
| CGI-S | 5.6  (0.5) | 5.5  (0.5) | *99.2*  *(3.6)* | 4.8  (0.9) | *86.5^**^*  *(16.3)* | *^**^* | 3.8  (1.2) | ***69.6^**^***  ***(22.9)*** | *^**^* |
| Acc. 2B | 0.65 (0.24) | 0.69 (0.20) | *116 (52.6)* | 0.78 (0.22) | *132.6^*^ (64.6)* | *^**^* | 0.78 (0.21) | ***137.1^*^ (71.2)*** |  |
| Acc. 0B | 0.88 (0.25) | 0.94 (0.15) | *151.3 (181.4)* | 0.98 (0.04) | *155.1^*^ (182.2)* |  | 0.98 (0.04) | ***153.1+ (169.6)*** |  |
| RT 2B | 547.5 (312.1) | 539.3 (286.5) | *123.5 (114.1)* | 467 (248.9) | *104.1 (72.3)* | *^+^* | 535.8 (313.9) | ***128.8 (131.4)*** |  |
| RT 0B | 557.2  (173.6) | 512.5 (126.7) | *93.7^*^ (9.6)* | 515.2 (150.9) | *94.3 (17)* |  | 533.3 (175.5) | ***96.9 (18.4)*** |  |

**Table S2. Symptom and Performance Changes along Antidepressant Treatment**

Variables are presented as mean (standard deviation). Units are arbitrary except RT (msec), Acc. or contrasts between different sessions displayed as in percent or percent change. Abbreviations: uncorrected *P* <0.005**; *P* <0.05*; *P* <0.10^+^; paired t-test relative to baseline/previous session (Italics); 2B, 2-back; 0B, 0-back; d, day; Acc., Accuracy; HAMD, Hamilton Depression Rating Scale; MADRS, Montgomery-Åsberg Depression Rating Scale; RT, Reaction Time

| Sesssion | **R^2^** | | | **R^2^ CV** | | | **CI_95_ CV** | | | **PRESS** | | |
| --- | --- | --- | --- | --- | --- | --- | --- | --- | --- | --- | --- | --- |
|  | **main** | main-motion | main+ clinical | **main** | main-motion | main+ clinical | **main** | main-motion | main+ clinical | **main** | main-motion | main+ clinical |
| **Multivariate (amPFC, dlPFC, dlPFC-amPFC, dlPFC-PCC, dlPFC-PL)** | | | | | | | | | | | | |
| day 0 | **68.1** | 65.7 | 73.3 | **37.6** | 34 | 48.4 | **6.6,67.5** | 4.6,65.1 | 15,74.6 | **12719** | 13688 | 12391 |
| day 1 | **75.4** | 76.3 | 76 | **55.4** | 51.4 | 34.5 | **22.1,78.7** | 17.9,76.4 | 4.8,65.3 | **12692** | 10621 | 18191 |
| day 28 | **52.5** | 52.3 | 61.8 | **8.4** | 9.2 | 31.8 | **0,40.1** | 0,41.4 | 3.5,63.4 | **19758** | 19617 | 17174 |
| day 56 | **39.7** | 40.1 | 45.9 | **4.5** | 5 | 3.2 | **0,33.7** | 0,34.8 | 0,31.1 | **30482** | 30580 | 29007 |
| Σ(day 0,1) | **77.8** | 78 | 78.7 | **63** | 61 | 47.9 | **31.4,83** | 28.8,81.9 | 14.6,74.3 | **11423** | 11462 | 12671 |
| Σ(day 0-28) | **81.8** | 82.7 | 82.6 | **31.2** | 24.1 | 54.5 | **3.2,62.9** | 0.8,57.2 | 21.1,78.2 | **9559** | 10444 | 10904 |
| Σ(day 0-56) | **82.9** | 81.8 | 83.7 | **39.8** | 49.4 | 65 | **8.1,69.1** | 16,75.3 | 34,84 | **7593** | 8258 | 8191 |
| **Univariate amPFC** | | | | | | | | | | | | |
| day 0 | **5.4** | 5.3 | 30.5 | **0.2** | 0.2 | 0.2 | **0,20.7** | 0,21.3 | 0,20.7 | **26242** | 26223 | 22146 |
| day 1 | **6.4** | 6.9 | 29.1 | **5.2** | 5.8 | 5.2 | **0,35.1** | 0,36.1 | 0,35.1 | **25491** | 24995 | 24884 |
| day 28 | **2.6** | 2.8 | 27.5 | **1.4** | 1.7 | 1.4 | **0,10.3** | 0,9.4 | 0,10.3 | **26910** | 26686 | 21544 |
| day 56 | **11.1** | 11.2 | 34.3 | **8.9** | 8.9 | 8.9 | **0,40.9** | 0,40.9 | 0,40.9 | **24911** | 24873 | 19752 |
| Σ(day 0,1) | **7.1** | 7.3 | 30.5 | **4.5** | 4.4 | 4.5 | **0,33.7** | 0,33.6 | 0,33.7 | **25144** | 25053 | 23110 |
| Σ(day 0-28) | **17.5** | 18.4 | 34.1 | **17.3** | 18.1 | 17.3 | **0,50.8** | 0,51.6 | 0,50.8 | **21209** | 20987 | 22339 |
| Σ(day 0-56) | **45** | 45.9 | 51.3 | **43** | 44 | 43 | **10.5,71.2** | 11.2,71.8 | 10.5,71.2 | **14496** | 14356 | 14941 |
| **Univariate dlPFC** | | | | | | | | | | | | |
| day 0 | **33.2** | 33.4 | 44.9 | **33.4** | 33.2 | 33.4 | **4.3,64.6** | 4.2,64.5 | 4.3,64.6 | **17477** | 19069 | 16897 |
| day 1 | **32.3** | 32.1 | 46.8 | **34.6** | 34 | 34.6 | **4.9,65.4** | 4.6,65.1 | 4.9,65.4 | **19154** | 19475 | 17114 |
| day 28 | **25.4** | 24.9 | 40.2 | **2.5** | 1.3 | 2.5 | **0,8** | 0,10.4 | 0,8 | **19461** | 17272 | 17242 |
| day 56 | **32** | 32.8 | 40.3 | **33.3** | 34.6 | 33.3 | **4.2,64.6** | 4.9,65.4 | 4.2,64.6 | **17454** | 13090 | 12511 |
| Σ(day 0,1) | **52.8** | 52.2 | 59.4 | **53** | 52.3 | 53 | **19.6,77.4** | 18.9,77 | 19.6,77.4 | **12900** | 13890 | 13162 |
| Σ(day 0-28) | **49** | 48.5 | 56.2 | **44.2** | 44.6 | 44.2 | **11.4,72** | 11.8,72.3 | 11.4,72 | **13807** | 13053 | 13162 |
| Σ(day 0-56) | **49** | 51.5 | 56.2 | **44.2** | 49 | 44.2 | **11.4,72** | 15.6,75 | 11.4,72 | **13807** | 15620 | 16897 |
| **Univariate dlPFC-amPFC** | | | | | | | | | | | | |
| day 0 | **31.8** | 31.5 | 49.2 | **26.6** | 26.2 | 26.6 | **1.4,59.3** | 1.3,59 | 1.4,59.3 | **17946** | 18008 | 14914 |
| day 1 | **49** | 49.5 | 55.7 | **46.9** | 47.5 | 46.9 | **13.8,73.8** | 14.1,74 | 13.8,73.8 | **13083** | 12979 | 12672 |
| day 28 | **32.8** | 32.5 | 56.1 | **31.1** | 30.9 | 31.1 | **3.2,62.9** | 3.1,62.7 | 3.2,62.9 | **17188** | 17274 | 12959 |
| day 56 | **24.7** | 24.8 | 36.2 | **18.1** | 17.9 | 18.1 | **0,51.6** | 0,51.4 | 0,51.6 | **19548** | 19523 | 19149 |
| Σ(day 0,1) | **50** | 50.2 | 58.9 | **51.1** | 51.3 | 51.1 | **17.6,76.3** | 17.8,76.4 | 17.6,76.3 | **13331** | 13263 | 12165 |
| Σ(day 0-28) | **59** | 59.2 | 68.6 | **60.8** | 61.2 | 60.8 | **28.5,81.8** | 28.9,81.9 | 28.5,81.8 | **11028** | 10951 | 9705 |
| Σ(day 0-56) | **59** | 54.4 | 68.6 | **60.8** | 55.8 | 60.8 | **28.5,81.8** | 22.5,79 | 28.5,81.8 | **11028** | 11684 | 9705 |
| **Univariate dlPFC-PCC** | | | | | | | | | | | | |
| day 0 | **14.2** | 15.2 | 37.3 | **1.1** | 0.1 | 1.1 | **0,25.3** | 0,19.9 | 0,25.3 | **21524** | 21185 | 17428 |
| day 1 | **39.2** | 36.2 | 47.7 | **33.9** | 32.5 | 33.9 | **4.5,64.9** | 3.8,63.9 | 4.5,64.9 | **15953** | 16399 | 15041 |
| day 28 | **17.5** | 20.4 | 45.4 | **14.8** | 17.5 | 14.8 | **0,48.2** | 0,50.9 | 0,48.2 | **21115** | 20431 | 16384 |
| day 56 | **18.1** | 18.6 | 31.6 | **1.6** | 0.8 | 1.6 | **0,9.7** | 0,12 | 0,9.7 | **20557** | 20685 | 19381 |
| Σ(day 0,1) | **36.2** | 34.8 | 48.7 | **32.4** | 30.9 | 32.4 | **3.8,63.9** | 3.1,62.7 | 3.8,63.9 | **16086** | 16428 | 14251 |
| Σ(day 0-28) | **38.7** | 38.3 | 53.7 | **37.1** | 36 | 37.1 | **6.4,67.3** | 5.7,66.4 | 6.4,67.3 | **15500** | 15649 | 13123 |
| Σ(day 0-56) | **38.7** | 34.9 | 53.7 | **37.1** | 33.4 | 37.1 | **6.4,67.3** | 4.3,64.6 | 6.4,67.3 | **15500** | 16538 | 13123 |
| **Univariate dlPFC-PL** | | | | | | | | | | | | |
| day 0 | **34.6** | 34.4 | 45.2 | **23.4** | 24.5 | 23.4 | **0.6,56.6** | 0.9,57.5 | 0.6,56.6 | **16533** | 16541 | 16654 |
| day 1 | **55.9** | 55.9 | 58.7 | **55.8** | 56.4 | 55.8 | **22.6,79** | 23.2,79.3 | 22.6,79 | **11437** | 11372 | 12470 |
| day 28 | **34** | 33.5 | 45.6 | **22.4** | 21.6 | 22.4 | **0.4,55.6** | 0.3,55 | 0.4,55.6 | **18038** | 18270 | 17002 |
| day 56 | **11.7** | 11.8 | 28.9 | **1.5** | 1.4 | 1.5 | **0,26.7** | 0,26.6 | 0,26.7 | **24940** | 24898 | 21540 |
| Σ(day 0,1) | **58.7** | 58.9 | 61.2 | **59.1** | 59.8 | 59.1 | **26.4,80.8** | 27.1,81.1 | 26.4,80.8 | **10746** | 10726 | 11334 |
| Σ(day 0-28) | **69.5** | 69.2 | 70.4 | **68.6** | 68.7 | 68.6 | **38.9,85.8** | 39.2,85.9 | 38.9,85.8 | **8152** | 8250 | 8679 |
| Σ(day 0-56) | **69.5** | 71.6 | 70.4 | **68.6** | 66.1 | 68.6 | **38.9,85.8** | 35.4,84.5 | 38.9,85.8 | **8152** | 7672 | 8679 |

**Table S3. Imaging Predictors of depression recovery – Main Model, Motion Residuals, With the Best Clinical Predictor (TN)**

Explained variance after incorporating all patients (R^2^) and based on leave-one-out cross-validation (R^2^ CV, CI, PRESS) for each mediator and prognostic predictor. Abbreviations: A, activation (2B-0B contrast), amPFC, anterior medial prefrontal cortex; dlPFC, dorsolateral PFC; PCC, posterior cingulate cortex; PL, parietal lobe; FCi, context-independent functional connectivity; FCd, context-dependent FC; CI, 95% Confidence Interval; CV, leave-one-out cross-validation; PRESS, Predicted Residual Error Sum of Squares; Q, quartile; R^2^, variance; TN, treatment naive

| **Mediator; 2B-0B Activation amPFC** | | | | |
| --- | --- | --- | --- | --- |
|  | day 0 | day 1 | day 28 | day 56 |
| day 0 |  | **0.59 (0.24,0.81)** | **0.59 (0.24,0.8)** | **0.55 (0.18,0.78)** |
| day 1 |  |  | **0.45 (0.06,0.73)** | **0.47 (0.08,0.74)** |
| day 28 |  |  |  | **0.6 (0.25,0.81)** |
| **Prognostic Predictor; 2B-0B Activation dlPFC** | | | | |
|  | day 0 | day 1 | day 28 | day 56 |
| day 0 |  | 0.24 (-0.18,0.59) | 0.32 (-0.1,0.65) | **0.68 (0.38,0.85)** |
| day 1 |  |  | **0.61 (0.27,0.82)** | 0.39 (-0.02,0.69) |
| day 28 |  |  |  | **0.46 (0.06,0.73)** |
| **Prognostic Predictor; Context-independent FC right dlPFC-amPFC** | | | | |
|  | day 0 | day 1 | day 28 | day 56 |
| day 0 |  | **0.61 (0.27,0.82)** | 0.3 (-0.12,0.63) | **0.48 (0.09,0.74)** |
| day 1 |  |  | **0.48 (0.09,0.74)** | **0.66 (0.34,0.84)** |
| day 28 |  |  |  | **0.54 (0.17,0.78)** |
| **Prognostic Predictor; Context-independent FC right dlPFC-PCC** | | | | |
|  | day 0 | day 1 | day 28 | day 56 |
| day 0 |  | 0.35 (-0.06,0.67) | **0.5 (0.12, 0.76)** | **0.58 (0.23,0.8)** |
| day 1 |  |  | 0.22 (-0.2,0.85) | **0.61 (0.27,0.82)** |
| day 28 |  |  |  | **0.53 (0.15,0.77)** |
| **Prognostic Predictor; Context-dependent (0B) FC right dlPFC-iPL** | | | | |
|  | day 0 | day 1 | day 28 | day 56 |
| day 0 |  | **0.46 (0.06,0.73)** | 0.32 (-0.1,0.65) | 0.01 (-0.4,0.42) |
| day 1 |  |  | 0.33 (-0.09,0.65) | 0.29 (-0.13,0.63) |
| day 28 |  |  |  | 0.18 (-0.24,0.55) |

**Table S4. Intra-Class-Correlations, ICC (3,1)**

ICC (3,1) to estimate the single measure consistency between scans. Values above 0.4 (bold) are interpreted as high consistency between measures. Abbreviations: 2B, 2-back, 0B; 0-back; FC, functional connectivity; amPFC, anterior-medial PFC; dlPFC, dorsolateral PFC; iPL, inferior parietal lobe; PCC, posterior cingulate cortex

| Session | All Patients | | | | Leave-One-Out Cross-Validation (CV) | | | |
| --- | --- | --- | --- | --- | --- | --- | --- | --- |
|  | Threshold | SP | SN | AUC | Threshold | SP | SN | AUC |
| Multivariate (amPFC, dlPFC, dlPFC-amPFC, dlPFC-PCC, dlPFC-PL) | | | | | | | | |
| day 0 | -1.22 | 0.82 | 1 | 0.93 | 1.62 | 0.82 | 1 | 0.92 |
| day 1 | 0.58 | 0.82 | 0.91 | 0.89 | 1.67 | 0.73 | 1 | 0.9 |
| day 28 | 0.84 | 1 | 0.82 | 0.91 | 2.33 | 1 | 0.73 | 0.87 |
| day 56 | 1.09 | 1 | 0.64 | 0.83 | 2.26 | 0.96 | 0.6 | 0.79 |
| Σ(day 0,1) | -1.84 | 0.82 | 1 | 0.93 | 1.48 | 0.82 | 1 | 0.93 |
| Σ(day 0,1,28) | -1.79 | 0.82 | 1 | 0.96 | 1.77 | 0.82 | 1 | 0.94 |
| Σ(day 0,1,28,56) | -1.79 | 0.82 | 1 | 0.96 | 1.77 | 0.82 | 1 | 0.94 |
| Univariate amPFC | | | | | | | | |
| day 0 | -0.39 | 0.45 | 0.91 | 0.61 | 0.47 | 0.45 | 0.91 | 0.58 |
| day 1 | 0.36 | 0.73 | 0.55 | 0.63 | 0.37 | 0.27 | 1 | 0.58 |
| day 28 | -0.13 | 0.64 | 0.64 | 0.53 | 0.5 | 0.74 | 0.74 | 0.77 |
| day 56 | -0.34 | 0.73 | 0.55 | 0.6 | 0.48 | 0.45 | 0.82 | 0.57 |
| Σ(day 0,1) | -0.62 | 0.45 | 0.91 | 0.6 | 0.48 | 0.48 | 0.87 | 0.54 |
| Σ(day 0,1,28) | 0.87 | 0.82 | 0.64 | 0.73 | 0.43 | 0.45 | 1 | 0.69 |
| Σ(day 0,1,28,56) | 0.36 | 0.73 | 0.91 | 0.83 | 0.54 | 0.69 | 0.96 | 0.82 |
| Univariate dlPFC | | | | | | | | |
| day 0 | -0.3 | 0.64 | 1 | 0.85 | 0.34 | 0.64 | 1 | 0.83 |
| day 1 | -0.52 | 0.73 | 0.91 | 0.72 | 0.41 | 0.73 | 0.91 | 0.69 |
| day 28 | 0.19 | 0.91 | 0.64 | 0.83 | 0.49 | 0.78 | 0.78 | 0.8 |
| day 56 | -0.76 | 0.55 | 1 | 0.79 | 0.27 | 0.55 | 1 | 0.79 |
| Σ(day 0,1) | -0.16 | 0.73 | 0.91 | 0.79 | 0.37 | 0.69 | 0.96 | 0.76 |
| Σ(day 0,1,28) | -1.06 | 0.73 | 1 | 0.83 | 0.31 | 0.73 | 1 | 0.81 |
| Σ(day 0,1,28,56) | -1.06 | 0.73 | 1 | 0.83 | 0.31 | 0.73 | 1 | 0.81 |
| Univariate dlPFC-amPFC | | | | | | | | |
| day 0 | 0.34 | 0.91 | 0.64 | 0.81 | 0.53 | 0.82 | 0.73 | 0.79 |
| day 1 | 0.52 | 0.82 | 0.82 | 0.86 | 0.39 | 0.73 | 0.91 | 0.83 |
| day 28 | 0.95 | 1 | 0.45 | 0.76 | 0.47 | 0.64 | 0.82 | 0.74 |
| day 56 | 0.44 | 1 | 0.55 | 0.81 | 0.69 | 1 | 0.55 | 0.8 |
| Σ(day 0,1) | 0.2 | 0.82 | 0.82 | 0.88 | 0.48 | 0.78 | 0.87 | 0.86 |
| Σ(day 0,1,28) | -0.07 | 0.82 | 0.91 | 0.9 | 0.4 | 0.78 | 0.96 | 0.88 |
| Σ(day 0,1,28,56) | -0.07 | 0.82 | 0.91 | 0.9 | 0.4 | 0.78 | 0.96 | 0.88 |
| Univariate dlPFC-PCC | | | | | | | | |
| day 0 | 0.04 | 0.82 | 0.64 | 0.73 | 0.51 | 0.82 | 0.64 | 0.72 |
| day 1 | 0.42 | 0.91 | 0.73 | 0.86 | 0.59 | 0.83 | 0.83 | 0.86 |
| day 28 | 0.76 | 1 | 0.45 | 0.79 | 0.59 | 0.78 | 0.69 | 0.74 |
| day 56 | -0.19 | 0.82 | 0.82 | 0.83 | 0.42 | 0.82 | 0.82 | 0.79 |
| Σ(day 0,1) | -0.33 | 0.82 | 0.91 | 0.87 | 0.41 | 0.82 | 0.91 | 0.85 |
| Σ(day 0,1,28) | -1.01 | 0.73 | 1 | 0.91 | 0.26 | 0.73 | 1 | 0.88 |
| Σ(day 0,1,28,56) | -1.01 | 0.73 | 1 | 0.91 | 0.26 | 0.73 | 1 | 0.88 |
| Univariate dlPFC-PL | | | | | | | | |
| day 0 | 0.14 | 1 | 0.73 | 0.93 | 0.59 | 1 | 0.73 | 0.9 |
| day 1 | 0.31 | 0.91 | 0.82 | 0.88 | 0.66 | 0.91 | 0.82 | 0.88 |
| day 28 | 0.66 | 0.91 | 0.64 | 0.83 | 0.47 | 0.69 | 0.87 | 0.77 |
| day 56 | 0.77 | 1 | 0.45 | 0.66 | 0.58 | 0.92 | 0.46 | 0.63 |
| Σ(day 0,1) | 0.1 | 1 | 0.91 | 0.98 | 0.61 | 1 | 0.91 | 0.97 |
| Σ(day 0,1,28) | 0.6 | 1 | 0.91 | 0.99 | 0.57 | 0.91 | 1 | 0.96 |
| Σ(day 0,1,28,56) | 0.6 | 1 | 0.91 | 0.99 | 0.57 | 0.91 | 1 | 0.96 |

**Table S5. Youden Index ROC Parameters, Predictive Performance for Remission**

ROC parameters calculated for each mediator and prognostic predictor with and without CV at an optimal trade-off threshold between sensitivity and specificity, as indicated by the Youden index(18). Abbreviations: A, activation (2B-0B contrast), amPFC, anterior medial prefrontal cortex; dlPFC, dorsolateral PFC; PCC, posterior cingulate cortex; PL, parietal lobe; CV, leave-one-out cross-validation; SP, specificity; SN, sensitivity; AUC, area under the curve
