# Supplementary figures and images for: Prefrontal networks dynamically related to recovery from major depressive disorder: a longitudinal pharmacological fMRI study
Source: Transl Psychiatry. 2019 Feb 4;9:64. doi: 10.1038/s41398-019-0395-8 (PMC6362173; doi:10.1038/s41398-019-0395-8)

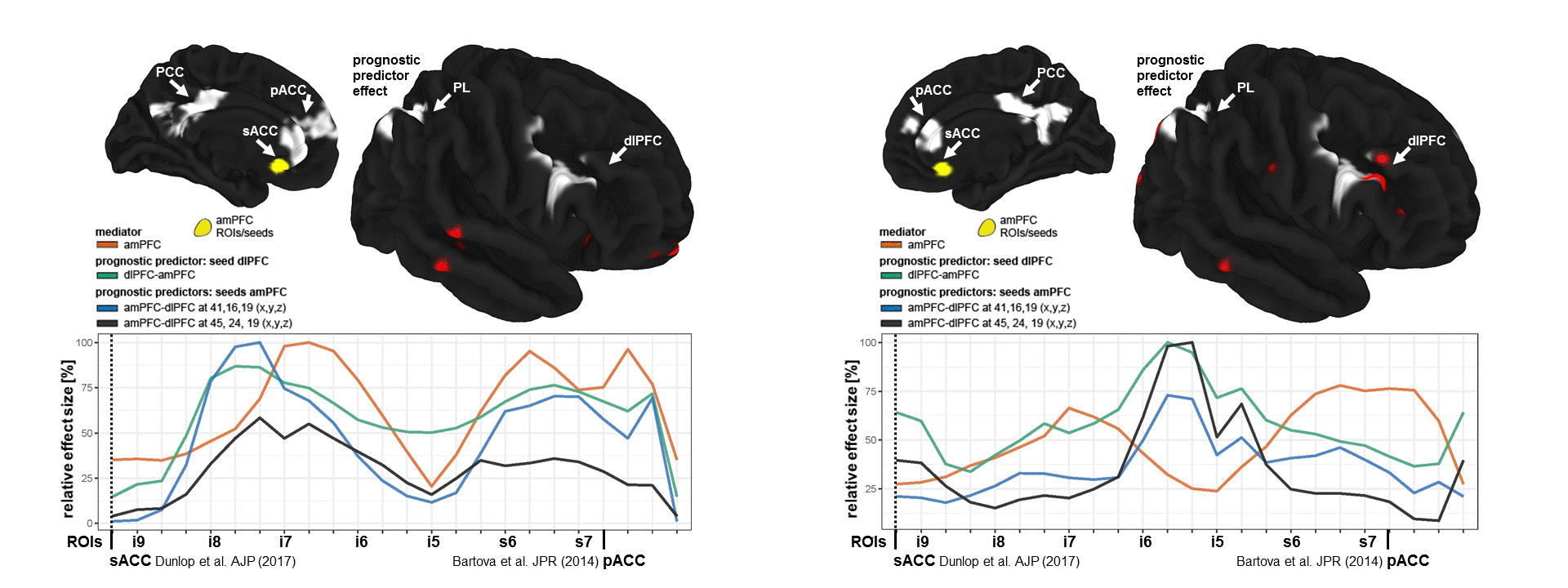

Supplement: Supplementary file 2 — Supplemental Video [file 41398_2019_395_MOESM2_ESM.gif]
